# Ripening-Associated Changes in Physicochemical Properties and Metabolomic Profiles of Processing Tomato

**DOI:** 10.3390/foods15193559

**Published:** 2026-10-08

**Authors:** Yankai Hou, Gianfranco Picone, Wanyue Li, Xueming Wang, Ruilin Yang, Jie Kang, Chenglin Zhu, Luca Laghi

**Affiliations:** 1 College of Pharmacy and Food, Southwest Minzu University, Chengdu 610041, China; 2 Department of Agricultural and Food Sciences, University of Bologna, 47521 Cesena, Italy; gianfranco.picone@unibo.it (G.P.); l.laghi@unibo.it (L.L.); 3 Xinjiang Guannong Testing Technology Co., Ltd., Korla 841000, China

**Keywords:** processing tomato, ripening stage, metabolomics, volatile compounds, quality evaluation

## Abstract

Understanding ripening-associated physicochemical and metabolic changes is essential for evaluating quality development and optimizing harvest maturity in processing tomatoes. In this study, the processing tomato cultivar “Zhongshu 5810” (*Solanum lycopersicum* L.) was investigated across six consecutive ripening stages using physicochemical analysis, LC-MS-MS, and HS-SPME-GC-MS-based metabolomics. During ripening, total soluble solids significantly increased, whereas γ-aminobutyric acid and total phenolic content significantly decreased (*p* < 0.05). GC–MS identified 77 volatile compounds, and the 20 volatile compounds with the highest VIP scores were selected as candidate markers of ripening-related aroma variation, revealing stage-dependent remodeling of volatile metabolism. LC-MS detected 4157 non-volatile features and revealed pronounced stage-dependent remodeling of the non-volatile metabolome, with distinct accumulation patterns among diverse chemical classes. Multivariate analysis and pathway enrichment analysis indicated that amino acid, nucleotide, cofactor, and lipid-related metabolism contributed substantially to ripening-associated metabolic variation. These findings provide a comprehensive characterization of quality-related metabolomic changes during processing tomato ripening and identify potential maturity-associated indicators for harvest optimization and quality management.

## 1. Introduction

Tomato (*Solanum lycopersicum* L.) is a major horticultural crop cultivated worldwide and an important raw material for the food-processing industry for both fresh consumption and processed products, including sauces, pastes, and juices. The quality of tomato is determined by the coordinated evolution of physicochemical properties and the concentration of primary metabolites, bioactive compounds, and volatile compounds during ripening. In particular, organic acids and soluble sugars contribute to the balance between acidity and sweetness, whereas volatile compounds determine aroma formation and consumer perception of flavor quality [1,2]. Moreover, phenolic compounds, carotenoids, and other phytochemicals contribute to the nutritional value and functional properties of tomato [3,4]. For processing tomatoes, the ripening stage is particularly important because variations in soluble solids content, acidity, color, and metabolite composition directly affect raw material quality and processing performance [5]. Therefore, comprehensive characterization of metabolic changes during tomato ripening is essential for evaluating maturity and optimizing harvest strategies for processing tomato production.

The ripening of tomato is a highly coordinated physiological process leading to extensive metabolome’s remodeling involving carbohydrate metabolism, amino acid metabolism, phenolic biosynthesis, and aroma formation [3,4]. Previous studies have demonstrated that ripening-associated metabolic changes are strongly dependent on developmental stage and genotype. For example, the γ-aminobutyric acid (GABA) content increases during early fruit development, reaches a relatively high level before or around the breaker stage, and subsequently decreases during later ripening because of enhanced GABA catabolism [6]. Metabolomic investigations have further revealed substantial changes in diverse compound classes, including organic acids, amino acids, phenolic acids, alkaloids, flavonoids, lipids, and nucleotides during tomato ripening. Antala et al. investigated phytochemical and metabolic changes among four tomato genotypes and demonstrated genotype- and stage-dependent variations in primary and specialized metabolites [7]. Pan et al. proposed an integration between transcriptomic and metabolomic approaches for the characterization of metabolic changes in cherry tomatoes during ripening, thus revealing extensive remodeling of flavor-related metabolites [8]. Yang et al. further demonstrated that ripening-related transcriptional regulation influenced the concentration of alkaloids, flavonoids and phenolic acids in tomato [9]. These studies indicate that tomato ripening involves complex compound-specific metabolic transitions rather than simple increases or decreases in individual quality components.

Volatile compounds are major contributors to tomato aroma and are closely associated with sensory quality. Tomato volatiles mainly originate from fatty acid, amino acid, phenylalanine, and carotenoid metabolism, generating diverse chemical classes including aldehydes, alcohols, ketones, esters, terpenoids, and aromatic compounds [10,11]. During ripening, tomato undergoes substantial changes in volatile composition, with early-stage green aroma compounds gradually replaced by fruity and floral aroma compounds during later stages [10,12,13]. Carotenoid-derived volatile compounds, like β-ionone and related apocarotenoids, contribute strongly to ripe tomato aroma and are closely associated with carotenoid metabolism [14]. Li et al. combined physicochemical analysis, UPLC–MS/MS, and HS-SPME–GC–MS to characterize antioxidant-related, volatile and non-volatile metabolites in cherry tomato along ripening, revealing enhanced accumulation of aroma-associated compounds at the red-ripe stage [15]. However, studies comparing only immature and fully ripe stages provide limited information regarding transient metabolic changes occurring during intermediate ripening periods.

Although considerable progress has been made in understanding tomato ripening, several limitations remain. First, many previous works studied fresh-market tomato or cherry tomato, rather than processing tomato cultivars, despite the distinct quality requirements of processing tomatoes. Second, most investigations have compared only two or three representative maturity stages, which may overlook gradual metabolomic transitions occurring throughout the ripening process. Third, although liquid chromatography–mass spectrometry (LC–MS) and gas chromatography–mass spectrometry (GC–MS) provide complementary analytical coverage of non-volatile and volatile metabolites, integrated analyses across continuous commercial ripening stages remain relatively limited. Jeong et al. applied combined GC–MS and LC–MS approaches to investigate tomato metabolomic changes; however, their study mainly focused on postharvest ripening regulation rather than natural maturation across commercial maturity stages [16]. Therefore, high-resolution characterization of metabolome trajectories throughout consecutive ripening stages is still needed, particularly for processing tomato cultivars used in industrial production.

In this study, the processing tomato cultivar “Zhongshu 5810” grown in Xinjiang, China, was chosen as the research material. Six consecutive stages of ripening, namely mature green, breaker, turning, pink, light red, and red-ripe stages, were systematically investigated by integrating physicochemical measurements, headspace solid-phase microextraction coupled with gas chromatography–mass spectrometry (GC-MS), and liquid chromatography–mass spectrometry (LC-MS)-based metabolomics. Unlike previous studies mainly focusing on fresh-market or cherry tomatoes and limited maturity comparisons, this study provides a detailed characterization of metabolic changes across ripening stages in a Xinjiang processing tomato cultivar throughout the complete ripening continuum. The objectives were to (i) characterize stage-dependent changes in physicochemical properties, (ii) identify dynamic variations in volatile and non-volatile metabolic profiles, and (iii) determine potential metabolites that could be used as indicators of maturity evolution. The findings provide insights into quality-related metabolomic changes during processing tomato ripening and provide a chemical basis for harvest optimization and quality management in tomato production at the industrial level.

## 2. Materials and Methods

### 2.1. Sample Collection

Fruits of the processing tomato cultivar “Zhongshu 5810” were collected from commercial production fields in Xinjiang, China, during the commercial harvest season (August–September 2025). Upon harvest, fruits were immediately brought to the laboratory and processed within 24 h. Fruits with uniform appearance and size and with no visible mechanical damage, disease symptoms, or physiological disorders were selected for subsequent analyses.

According to the USDA tomato color classification system and previously reported criteria [17,18], fruits were classified into six consecutive stages of ripening based on external color: mature green (MG), breaker (BR), turning (T), pink (P), light red (LR), and red ripe (R). For each ripening stage, fruits were randomly assigned to biological replicates. Each biological replicate consisted of a pooled sample prepared from multiple fruits at the same maturity stage. Four biological replicates were used for all physicochemical, biochemical, GC–MS, and LC–MS analyses.

After arrival at the laboratory, fruits were cleaned with distilled water and gently dried. Peduncles and sepals were removed, and fruits were cut into small pieces, then homogenized with a laboratory blender. Fresh homogenates were immediately used for pH and total soluble solids determination. The remaining material was rapidly frozen in liquid nitrogen and maintained at −80 °C until analysis. Repeated freeze–thaw cycles were avoided throughout sample preparation.

### 2.2. Determination of pH

The pH was measured directly in freshly prepared tomato homogenate with a digital pH meter (Shanghai INESA Scientific Instrument Co., Ltd., Shanghai, China). Before measurement, a calibration of the instrument was performed with standard buffer solutions at pH 4.00, 6.86, and 9.18. Between each measurement, the electrode was rinsed with ultrapure water and then dried through gentle blotting. pH values were then recorded after stabilization.

### 2.3. Determination of Soluble Solids

Total soluble solids (TSS) was quantified using a digital refractometer by following the Official Method 932.12 from AOAC [19]. The refractometer (r2mini, Reichert Technologies, Depew, NY, USA) was calibrated with distilled water before use. The prism was covered with freshly prepared tomato juice, and the result was expressed as °Brix.

### 2.4. Determination of Total Phenolics

Total phenolic content (TPC) was quantified using the Folin–Ciocalteu method, with minor modifications [20]. Approximately 50 mg of freeze-dried tomato powder was added to 80% (*v*/*v*) aqueous methanol (1.0 mL) and then sonicated for 20 min. Centrifugation at 14,000 rpm for 15 min was then conducted using a refrigerated high-speed centrifuge (TGL-1650, Sichuan Shuke Instrument Co., Ltd., Chengdu, China). The resulting supernatant (20 μL) was sequentially added to 790 μL of distilled water, 50 μL of Folin–Ciocalteu reagent, and 150 μL of 20% (*w*/*v*) sodium carbonate solution. After 1 h in the dark at room temperature, absorbance was measured at 765 nm with a UV–visible spectrophotometer. The reference standard selected was gallic acid. The results were presented as milligrams of gallic acid equivalents per gram of dry weight (mg GAE g^−1^ DW).

### 2.5. Determination of GABA

The γ-aminobutyric acid (GABA) content was quantified by following a previously reported method, slightly adjusted [6]. Freeze-dried tomato powder (250 mg) was added to 10 mL of 70% (*v*/*v*) aqueous ethanol, followed by three consecutive 10 min sonication cycles. After centrifugation at 5700× *g* for 10 min at room temperature, 1 mL of the supernatant was passed through a 0.22 μm membrane filter. The filtrate was analyzed using a Biochrom 30+ amino acid analyzer (Biochrom Ltd., Cambridge, UK) equipped with an H-1649 lithium accelerated resin column and a post-column ninhydrin derivatization system. GABA was detected at 570 nm and identified by comparing its retention time with an authentic standard. An external calibration curve was used for quantification, while the results were given in milligrams per gram of dry weight (mg g^−1^ DW).

### 2.6. GC-MS Analysis

Headspace solid-phase microextraction, coupled with gas chromatography–mass spectrometry (HS-SPME-GC-MS), was used to analyze volatile compounds by following a previously reported method, slightly modified [21]. Homogenized tomato sample (1.5 g) was transferred to a headspace vial of 20 mL and equilibrated at 50 °C for 15 min. Subsequently, a 50/30 μm divinylbenzene/carboxen/polydimethylsiloxane (DVB/CAR/PDMS) fiber was exposed to the headspace. This was done at 50 °C for 30 min under continuous agitation at 300 rpm. Then, the fiber was swiftly introduced into the injection port of an ISQ 7610 GC-MS system (Thermo Fisher Scientific, Waltham, MA, USA) and desorbed at 230 °C in splitless mode. Volatile compounds were separated on a TG-WaxMS B capillary column (30 m × 0.25 mm i.d., 0.25 μm film thickness) using highly pure helium as the carrier gas at a constant flow rate of 1.0 mL min^−^^1^. The temperature of the oven was initially kept at 45 °C for 3 min, increased to 230 °C at 5 °C min^−1^, and maintained at 230 °C for 5 min. Mass spectra were obtained in electron ionization mode at 70 eV over an *m*/*z* range of 35–500. Compounds were identified when the match score exceeded 700 in the NIST 11 library. The relative abundances of all volatile compounds were calculated by peak-area normalization and expressed as percentages of the total volatile peak area.

### 2.7. LC-MS Analysis

Ultra-performance liquid chromatography, coupled with quadrupole time-of-flight mass spectrometry (UPLC–QTOF–MS), was used to analyze non-volatile metabolites, as described in a previously reported method, slightly modified [22]. Briefly, approximately 0.50 g of freeze-dried tomato powder was accurately weighed into a stoppered conical flask and extracted with 20 mL of methanol by sonication for 30 min. After cooling to room temperature, the flask was reweighed, and any solvent loss was compensated with methanol. The extract was thoroughly mixed, filtered through a 0.22 μm membrane, and transferred to an autosampler vial for analysis. To obtain a pooled quality-control (QC) sample, equal aliquots of all sample extracts were combined and injected periodically throughout the analytical sequence. This allowed evaluation of the stability of the instrument and analytical reproducibility. Chromatographic separation was performed using an ACQUITY UPLC H-Class system (Waters Corporation, Milford, MA, USA) equipped with an ACQUITY UPLC HSS T3 column (2.1 × 100 mm, 1.8 μm). The mobile phases consisted of 0.1% formic acid in water (A) and acetonitrile (B), and the gradient program was as follows: 0–2 min, 5% B; 2–12 min, 5–30% B; 12–18 min, 30–80% B; 18–22 min, 80–100% B; 22–25 min, 100% B; 25–25.1 min, 100–5% B; and 25.1–28 min, 5% B. The flow rate was maintained at 0.20 mL min^−1^, the column temperature was set at 30 °C, and the injection volume was 2.0 μL. Mass spectrometric detection was performed using a TripleTOF 5600 mass spectrometer (SCIEX, Framingham, MA, USA) equipped with a TurboIonSpray electrospray ionization source. Data were acquired separately in positive- and negative-ion modes over an *m*/*z* range of 50–2000, with an accumulation time of 1 s per scan. The ion-source parameters were set as follows: curtain gas, 35 psi; nebulizer gas, 50 psi; auxiliary gas, 50 psi; source temperature, 500 °C; and ion-spray voltage, 5000 V in positive-ion mode and −4500 V in negative-ion mode.

### 2.8. Statistical Analysis

Four biological replicates were analyzed for physicochemical measurements and GC-MS and LC-MS analyses. All data were expressed as mean ± standard deviation. SPSS was used to perform one-way ANOVA, followed by Duncan’s multiple range test (*p* < 0.05). Multivariate statistical analysis and metabolomics data visualization were performed using R software (Version 4.6.1), the MetaboAnalyst platform, and the Metware Cloud platform. Differentially accumulated metabolites were screened using VIP > 1, |log_2_FC| ≥ 1, and adjusted *p* < 0.05 after false discovery rate correction.

## 3. Results

### 3.1. Physicochemical Changes During Tomato Ripening

To characterize the physiological changes associated with tomato fruit maturation, the four representative physicochemical parameters, pH, TSS, TPC, and GABA, were analyzed during ripening. These allowed us to follow the changes in acidity, sweetness-related characteristics, phenolic compounds, and amino acid metabolism during fruit maturation [23,24]. The results provide fundamental information regarding quality evolution and facilitate the interpretation of subsequent metabolic changes.

As shown in Figure 1, the physicochemical properties of tomato varied distinctly among ripening stages. The pH values remained relatively stable from the MG to LR stages, ranging from 4.16 to 4.20, but increased significantly to 4.26 at the R stage (Figure 1a). The increase in pH during late ripening may be associated with the gradual reduction in organic acids, such as citrate and malate, which are consumed as respiratory substrates during fruit maturation [25]. In contrast, TSS increased continuously throughout ripening, from 2.61 °Brix at MG to 5.32 °Brix at R (Figure 1c). The increase in TSS indicates enhanced soluble sugar accumulation during fruit maturation, which is mainly attributed to starch degradation and the conversion of carbohydrates into soluble sugars [25,26]. The TSS level at the R stage was comparable to previously reported values for ripe tomato [27], suggesting that sugar accumulation was largely completed during the late ripening stage.

TPC and GABA exhibited decreasing trends during tomato ripening (Figure 1b,d). The decline in TPC may result from the transformation and utilization of phenolic compounds during fruit maturation, whereas the reduction in GABA suggests dynamic regulation of amino acid metabolism throughout the ripening process. Similar changes in phenolic compounds and GABA metabolism have been reported during tomato fruit maturation, indicating that secondary and primary metabolic pathways undergo coordinated adjustments during ripening [12,24].

Overall, tomato fruit ripening was characterized by increased TSS accompanied by decreased TPC and GABA contents, together with a slight increase in pH at the fully ripe stage. These coordinated changes indicate that fruit maturation involves extensive metabolomic adjustments affecting both primary and secondary metabolites. Therefore, GC–MS- and LC–MS-based metabolomic analyses were further performed to comprehensively investigate the changes in metabolism associated with tomato ripening.

### 3.2. Volatile Metabolite Remodeling During Tomato Ripening

A total of 77 volatile compounds were detected in “Zhongshu 5810” tomato across the six ripening stages, including 18 aldehydes, 17 alcohols, 10 terpenes, 9 ketones, 5 aromatic hydrocarbons, 4 esters, 4 phenols, 2 acids, and 8 compounds assigned to other chemical classes. Aldehydes and alcohols were the most diverse volatile groups, together accounting for 35 compounds and 44.9% of all detected volatile compounds. These two classes included many compounds contributing to the green, fresh, fruity, and ripe aroma characteristics of tomato fruit [10,12]. Tomato aroma is determined by a complex mixture of lipid-, amino acid-, and carotenoid-derived volatiles, and their relative abundance changes considerably during fruit development and ripening [10,13,28].

Stage-associated VOC occurrence was examined using a flower plot (Figure 2a). A compound was considered present at a given stage when it was detected in at least two of the four biological replicates. According to this criterion, tentatively identified 1-octen-3-ol was the only volatile compound detected across all six stages. In addition, stage-specific volatile compounds were detected as follows: 7 at MG, 3 at BR, 1 at T, 7 at P, 2 at LR, and 11 at R. The R stage contained the largest number of stage-specific volatile compounds, indicating increased diversification of the detectable volatile profile at full ripeness. These stage-specific distributions further reflected the dynamic remodeling of volatile metabolism throughout tomato maturation.

Marked modifications were observed in the relative contribution of the nine volatile classes during ripening (Figure 2b). At the MG stage, aromatic hydrocarbons and terpenes were predominant, accounting, respectively, for 42.5% and 32.0% of the total relative peak area. Aromatic hydrocarbons remained the largest class at the BR stage, representing 36.9%, whereas aldehydes, esters, and alcohols accounted for 18.4%, 15.6%, and 11.9%, respectively. A substantial compositional transition occurred at the T stage, when ketones increased to 37.1% and became the predominant volatile class. Ketones remained dominant at the P, LR, and R stages, accounting for 43.8%, 35.3%, and 37.8%, respectively. Aldehydes reached their highest proportion at the P stage, representing 30.9% of the total relative peak area, while alcohols increased during late ripening and accounted for 20.1% and 22.9% at the LR and R stages, respectively. In contrast, aromatic hydrocarbons decreased from 42.5% at MG to 2.1% at R, and terpenes declined from 32.0% at MG to approximately 1% at the LR and R stages. These results indicate that the volatile profile shifted from an aromatic hydrocarbon- and terpene-rich composition during early ripening to one characterized by higher proportions of ketones, aldehydes, and alcohols during middle and late ripening.

PCA was performed to obtain an overall picture of the differences in volatile composition among the six ripening stages (Figure 2c). PC1 and PC2 explained 35.9% and 17.2% of the total variance, respectively, for a cumulative explained variance of 53.1%. Biological replicates from the same stage generally clustered closely, indicating good within-stage reproducibility. MG and BR samples appeared mainly on the negative side of PC1 and exhibited relatively similar volatile profiles, whereas R samples showed markedly positive PC1 scores. LR samples were also distributed on the positive side of PC1 but remained separated from R samples along PC2. T and P samples showed intermediate distributions between immature and fully ripe stages. This distribution indicates a progressive transition in volatile composition from immature to fully ripe fruits and that intermediate ripening stages represent distinct phases of volatile development. Pronounced differences between immature green and red-ripe tomatoes have also been reported previously [28,29,30].

To identify the volatile compounds associated most strongly with ripening-related variation, VIP analysis was performed, and the 20 compounds with the highest VIP values were selected (Figure 2d). The selected compounds mainly belonged to alcohols, esters, ketones, and terpenes, suggesting that these volatile classes contributed strongly to the discrimination of different ripening stages. Alcohols and esters were the predominant groups. Representative compounds included hexanal, (E)-2-octenal, limonene, methyl salicylate, α-copaene, and geranylacetone. In addition, other important volatile contributors associated with ripening were also identified. These results suggest that lipid-derived oxidation products, phenylpropanoid/benzenoid volatiles, and terpene-related compounds were closely associated with aroma evolution during tomato ripening.

The observed changes were broadly consistent with the established biochemical origins of tomato volatiles. Fatty-acid-derived C5 and C6 compounds are generated mainly through the lipoxygenase pathway, with TomloxC participating in the formation of these volatiles [29,30]. C6 aldehydes and their corresponding alcohols are commonly associated with green, grassy, leafy, and fresh sensory characteristics of tomato fruit [10,13,31]. Phenylalanine-derived compounds, including phenylacetaldehyde and 2-phenylethanol, contribute floral, honey-like, and sweet notes, whereas carotenoid-derived compounds such as β-ionone and 6-methyl-5-hepten-2-one are associated with floral, fruity, and tomato-like aroma attributes [3,4,32]. Therefore, the decline in terpenes and aromatic hydrocarbons, together with the increased relative contribution of ketones, aldehydes, and alcohols, reflects extensive remodeling of the volatile profile during ripening.

Because the data were expressed as relative peak areas, these changes represent shifts in volatile composition rather than absolute changes in VOC concentrations. Overall, the results demonstrate a clear ripening-dependent transition in the VOC profile of “Zhongshu 5810”, from an early-stage profile rich in aromatic hydrocarbons and terpenes to a middle- and late-stage profile dominated by ketones, aldehydes, and alcohols.

### 3.3. Non-Volatile Metabolite Remodeling

#### 3.3.1. Stage-Dependent Separation of Metabolomic Profiles

Untargeted LC–MS analysis detected 4157 metabolic features in “Zhongshu 5810” tomato fruit across the six ripening stages. Based on database-assisted annotation, the major chemical classes were lipids and lipid-like molecules (18.2%), phenylpropanoids and polyketides (17.8%), alkaloids and derivatives (14.3%), amino acids and derivatives (12.8%), and terpenoids (12.3%). The remaining annotated features were mainly assigned to organic acids and derivatives, carbohydrates and carbohydrate conjugates, steroids and steroid derivatives, and other chemical classes. The broad distribution of these compounds is consistent with the extensive remodeling of primary and specialized metabolism during tomato fruit development and ripening [9,33,34]. In particular, phenylpropanoid- and terpenoid-related metabolites contribute to fruit color, antioxidant properties, and flavor development, whereas amino acids and organic acids are important determinants of nutritional and sensory quality [33,34]. Alkaloid-related features were also highly represented, in agreement with the marked conversion of bitter steroidal glycoalkaloids into less bitter derivatives during tomato ripening [9,34]. Because compound assignments were obtained using untargeted LC–MS, metabolite identities not confirmed using authentic standards were considered as putative annotations.

PCA revealed pronounced stage-dependent changes in the overall metabolic profiles (Figure 3a). PC1 and PC2 explained, respectively, 53.7% and 8.5% of the total variance, accounting for a cumulative contribution of 62.2%. Biological replicates from the same stage clustered closely, indicating good reproducibility. MG, BR, and T samples occupied relatively distinct regions of the score plot, whereas P, LR, and R were positioned close to one another on the positive side of PC1. These results suggest that the major metabolic transition occurred during early-to-middle ripening, approximately corresponding to the pink stage. Consistently, the heatmap showed that one group of metabolomic features was relatively abundant at MG and BR, whereas another group accumulated mainly at P, LR, and R, with T generally exhibiting an intermediate profile (Figure 3b). Similar stage-dependent redistribution of primary and specialized metabolites has been reported during tomato and cherry tomato ripening [4,25,26].

#### 3.3.2. Identification of Key Metabolomic Features by PLS-DA and VIP Analysis

Despite the limited number of samples, PLS-DA could offer further exploratory information on how the metabolome of tomato evolved during maturation. Therefore, to further refine the observation of metabolic features mostly contributing to discrimination among ripening stages, a partial least squares discriminant analysis (PLS-DA) model was calculated on normalized LC–MS data. Variable importance in projection (VIP) scores was subsequently calculated to evaluate the contribution of individual features to stage separation. Features with higher VIP values were considered to contribute more strongly to metabolome differentiation during tomato fruit ripening. The top 20 metabolic features ranked by VIP scores showed distinct relative abundance profiles across the six ripening stages (Figure 4; Table S3). Several features displayed early-stage enrichment, whereas others showed preferential accumulation during middle or late ripening stages, indicating that different groups of metabolites contributed differently to stage discrimination. These results suggest that the transition from immature green fruit to fully ripe fruit is accompanied by coordinated changes in several metabolic components, including amino acid derivatives, phenylpropanoid-related compounds, and other specialized metabolites.

#### 3.3.3. Correlation Patterns of Differentially Accumulated Metabolites

To further explore coordinated metabolic changes during ripening, correlation analysis was performed among representative differentially accumulated metabolic features. Twenty metabolites were selected based on their statistical significance and distinct ripening-associated accumulation patterns, and Pearson correlation coefficients were calculated using their relative abundance values across all samples (Figure 5).

Correlation analysis revealed two major co-accumulation patterns among the selected metabolites (Figure 5). A group of metabolites exhibited predominantly positive correlations with each other and showed higher abundance during early ripening stages. In contrast, another metabolite cluster displayed coordinated accumulation during middle-to-late ripening and showed mainly negative correlations with early-stage-associated metabolites. Representative late-ripening-associated metabolites, including pyroglutamic acid and 1-aminopropenal acetic acid, showed similar upward trends during fruit maturation, whereas several early-stage-enriched metabolites exhibited opposite accumulation patterns.

These correlation patterns indicate coordinated metabolomic redistribution during tomato ripening, with distinct metabolite modules associated with different developmental stages [4]. However, these correlations represent similarities in accumulation patterns rather than direct biochemical conversion or regulatory interactions. Complete annotations and LC–MS information for the selected metabolites are provided in Table S6.

#### 3.3.4. KEGG Pathway Enrichment of Differentially Accumulated Metabolites

Kyoto Encyclopedia of Genes and Genomes (KEGG) pathway enrichment analysis was based on the identified differentially accumulated metabolites to identify metabolic pathways associated with tomato ripening (Figure 6). Pathways that were enriched mainly related to amino acid metabolism, nucleotide metabolism, cofactor metabolism, and lipid-associated metabolism. Amino acid-related pathways were prominently enriched, including amino acid biosynthesis; glycine, serine, and threonine metabolism; alanine, aspartate, and glutamate metabolism; β-alanine metabolism; phenylalanine, tyrosine, and tryptophan biosynthesis; arginine and proline metabolism; tyrosine metabolism; and branched-chain amino acid biosynthesis. In addition, nucleotide metabolism, purine metabolism, pyrimidine metabolism, nicotinate and nicotinamide metabolism, and linoleic acid metabolism were also significantly enriched.

The enrichment of amino acid-related pathways suggests potential alterations in nitrogen metabolism during tomato fruit maturation [4]. Amino acids not only contribute directly to nutritional and sensory characteristics but also serve as precursors for the biosynthesis of organic acids, phenolic compounds, alkaloids, and aroma-related metabolites [8,33,34]. Enrichment of nucleotide-related pathways suggests potential changes in nucleotide metabolism during developmental transitions. Furthermore, enrichment of nicotinate and nicotinamide metabolism suggests potential changes in related metabolic processes during ripening [35]. The enrichment of lipid-associated pathways suggests potential changes in lipid metabolism during ripening. Collectively, these findings suggest that tomato ripening involves coordinated changes across multiple interconnected metabolic pathways rather than alterations in a single metabolic process.

### 3.4. Integrated Metabolomic Transition During Ripening

The integrated physicochemical, volatile, and non-volatile metabolic analyses revealed coordinated changes during the ripening of “Zhongshu 5810” tomato fruit. The progressive increase in total soluble solids (TSS), together with the decline in γ-aminobutyric acid (GABA) and total phenolic content (TPC), reflected substantial adjustments in carbohydrate accumulation, amino acid metabolism, and phenolic metabolism during fruit maturation. These physicochemical changes were further supported by metabolomic analyses, which revealed extensive changes in both volatile and non-volatile metabolomic profiles across ripening stages.

GC–MS analysis revealed a clear transition in volatile composition from an early-stage profile associated with green and immature characteristics to middle- and late-stage profiles, containing a greater proportion of aroma-related compounds. The relative contribution of aromatic hydrocarbons and terpenes decreased during ripening, whereas aldehydes, ketones, and alcohols became increasingly prominent at later stages, indicating progressive remodeling of aroma-associated metabolic pathways. These changes are consistent with the transition in tomato flavor from green notes toward more mature fruity and floral attributes [36].

LC–MS-based metabolomics further revealed extensive changes in non-volatile metabolism, with distinct stage-associated accumulation patterns among diverse chemical classes. Multivariate analysis and VIP-based feature selection identified key metabolic features contributing to discrimination among ripening stages, while correlation analysis revealed coordinated accumulation patterns among representative differentially accumulated metabolites. KEGG enrichment analysis indicated that ripening-associated metabolic changes were mainly associated with amino acid metabolism, nucleotide metabolism, cofactor metabolism, and lipid-related pathways, suggesting possible changes in primary and specialized metabolism during fruit maturation [33].

Collectively, the transition from the turning to the pink stage appeared to represent a major period of metabolic reorganization, during which substantial changes occurred in both aroma formation and non-volatile metabolic networks. These findings provide a comprehensive view of the metabolic modifications that occur in processing tomato fruit during ripening and establish a foundation for further identification of maturity-associated indicators relevant to harvest optimization and processing quality evaluation.

## 4. Conclusions

In this study, the dynamic changes in physicochemical characteristics, volatile compounds, and non-volatile metabolites in processing tomato cultivar “Zhongshu 5810” were systematically characterized across six consecutive ripening stages by integrating physicochemical measurements, GC–MS, and LC–MS-based metabolomics. Ripening was accompanied by a progressive increase in total soluble solids, together with significant decreases in total phenolic content and γ-aminobutyric acid, indicating coordinated adjustments in primary and secondary metabolism. GC–MS analysis identified 77 volatile compounds and revealed pronounced stage-dependent remodeling of aroma-related metabolism, characterized by a transition from early-stage volatile profiles enriched in aromatic hydrocarbons and terpenes toward later-stage profiles with increased contributions from aldehydes, ketones, and alcohols. LC–MS-based metabolomics detected 4157 metabolic features and demonstrated extensive reorganization of the non-volatile metabolome. Multivariate analysis, VIP-based feature selection, correlation analysis, and KEGG enrichment collectively suggested coordinated changes in metabolic networks involving amino acid, nucleotide, cofactor, and lipid-related pathways during ripening.

Overall, the transition from the turning to the pink stages represented a key period of metabolic reorganization, during which substantial changes occurred in both volatile and non-volatile metabolic networks. These findings provide a comprehensive understanding of quality-related metabolite trajectories in processing tomato and offer potential indicators for maturity evaluation and harvest optimization. Targeted quantification, sensory evaluation, and processing performance analyses are required to validate the practical significance of these candidate indicators.

## Figures and Tables

**Figure 1:**
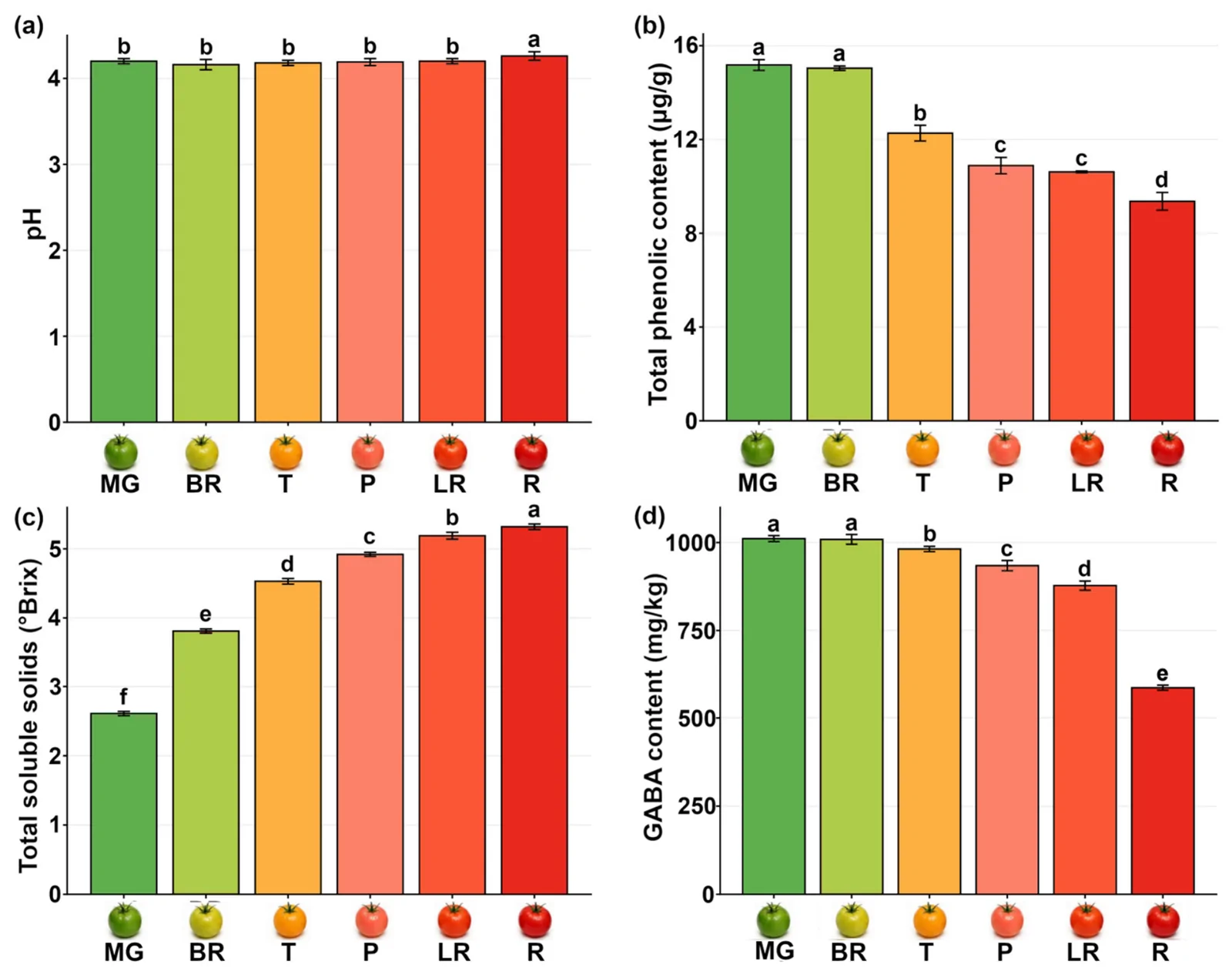
Physicochemical parameters evolution during tomato ripening: (**a**) pH; (**b**) TPC; (**c**) TSS; (**d**) GABA. Different letters highlight significant differences among ripening stages (*p* < 0.05).

**Figure 2:**
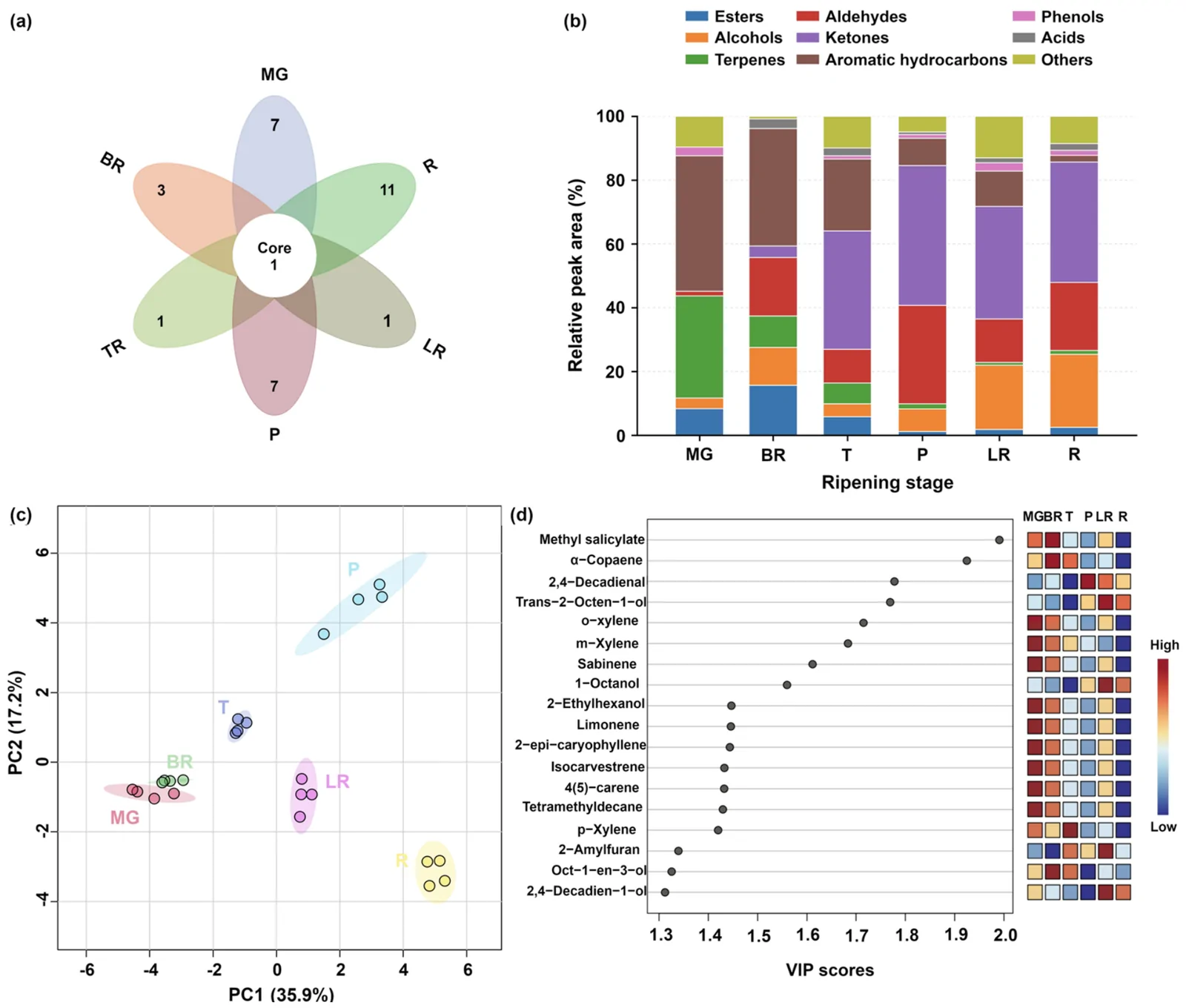
GC–MS-based profiling of volatile compounds in tomato cultivar “Zhongshu 5810” during ripening. (**a**) Flower plot showing shared and stage-specific volatile compounds; (**b**) stacked bar chart showing the relative integrated peak-area proportions of the nine volatile classes; (**c**) principal component analysis (PCA) score plot; and (**d**) VIP-ranked top 20 volatile compounds associated with tomato ripening.

**Figure 3:**
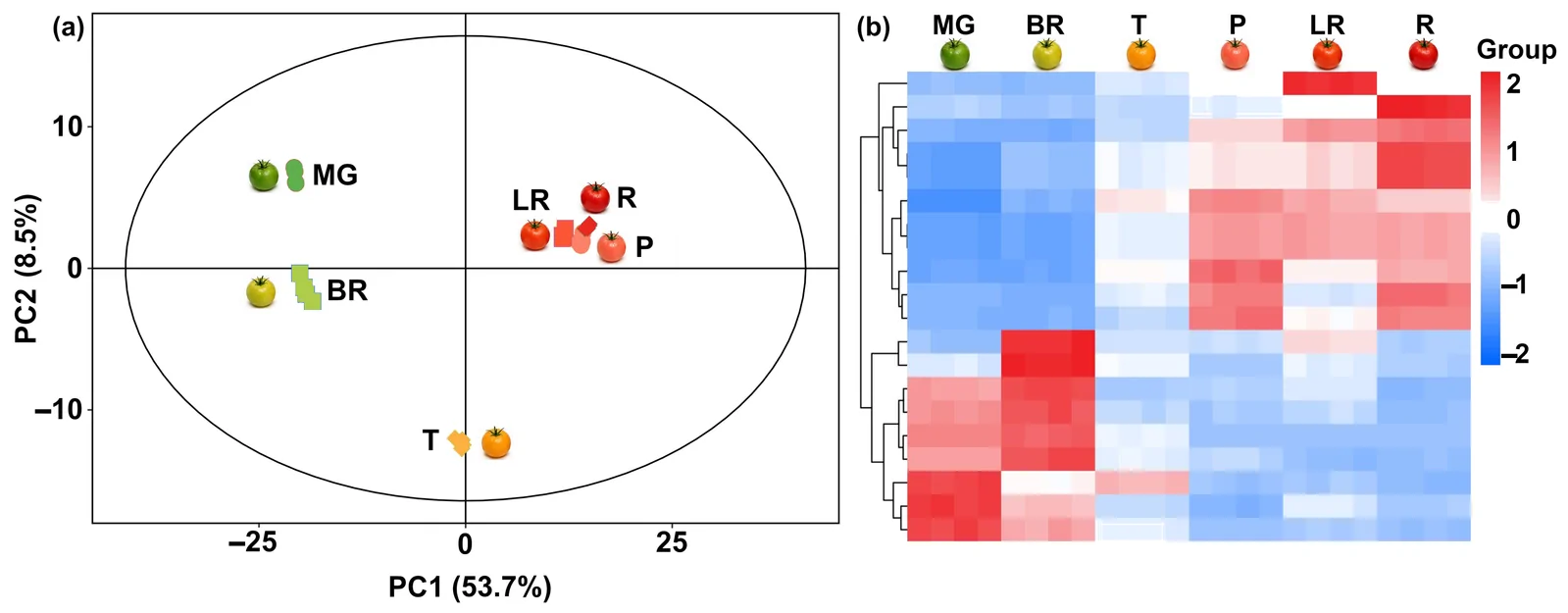
LC–MS-based metabolic profiles of “Zhongshu 5810” tomato fruit during ripening. (**a**) PCA score plot of normalized metabolomic features. The ellipse represents the 95% Hotelling’s T^2^ confidence region. (**b**) Heatmap of selected differentially accumulated metabolomic features across ripening stages.

**Figure 4:**
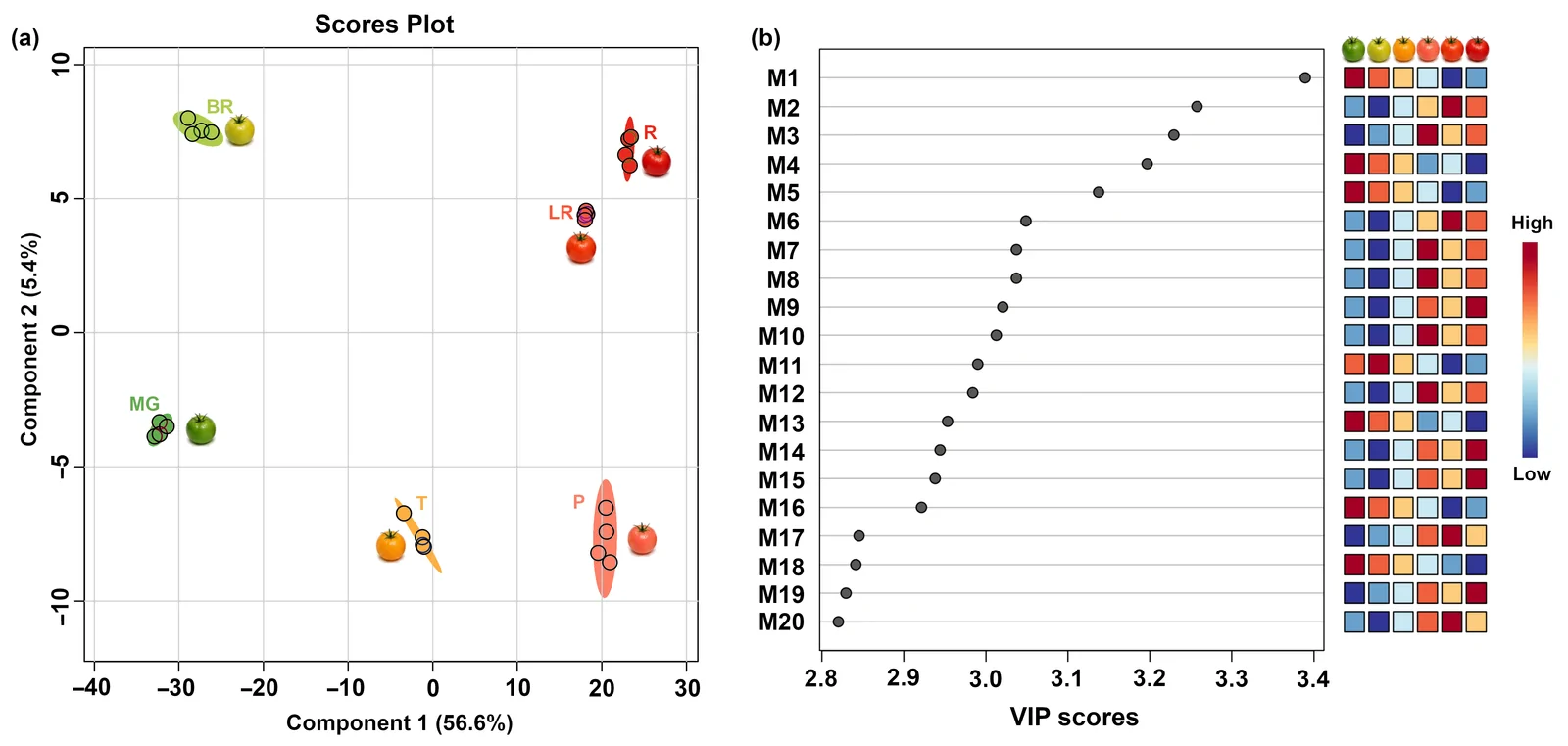
(**a**) Identification of key metabolic features based on VIP scores derived from PLS-DA during tomato fruit ripening. (**b**) The dot plot shows the VIP values of the top 20 metabolic features. The heatmap indicates their relative abundance patterns across six ripening stages (**b**). The annotations corresponding to M1–M20 are provided in Table S3.

**Figure 5:**
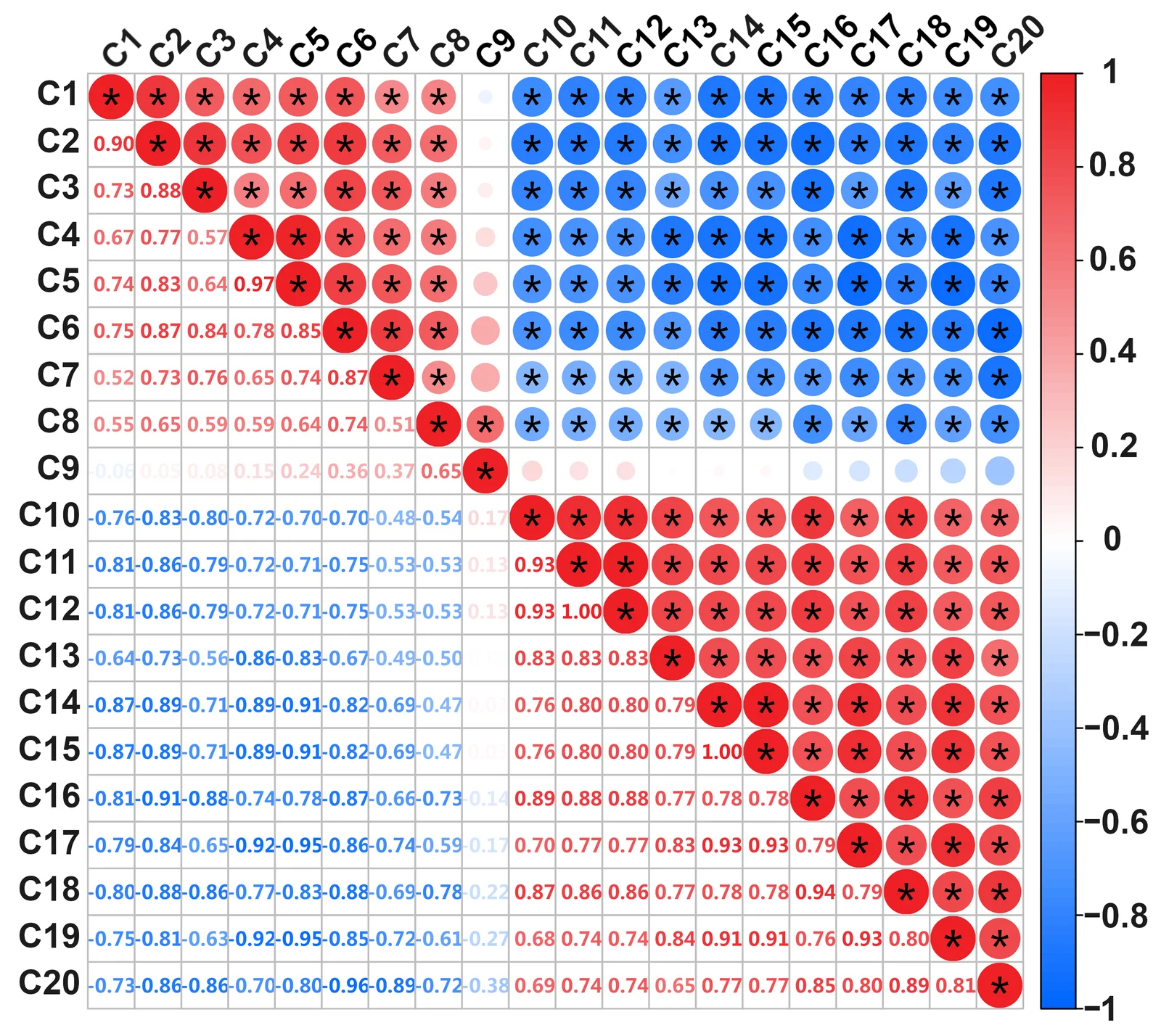
Pearson correlation matrix of representative differentially accumulated metabolic features during tomato ripening. Blue and red indicate, respectively, negative and positive correlations, while circle size represents the absolute correlation coefficient. Statistically significant correlations (*p* < 0.05) are indicated by asterisks. The annotations corresponding to C1–C20 are provided in Table S4.

**Figure 6:**
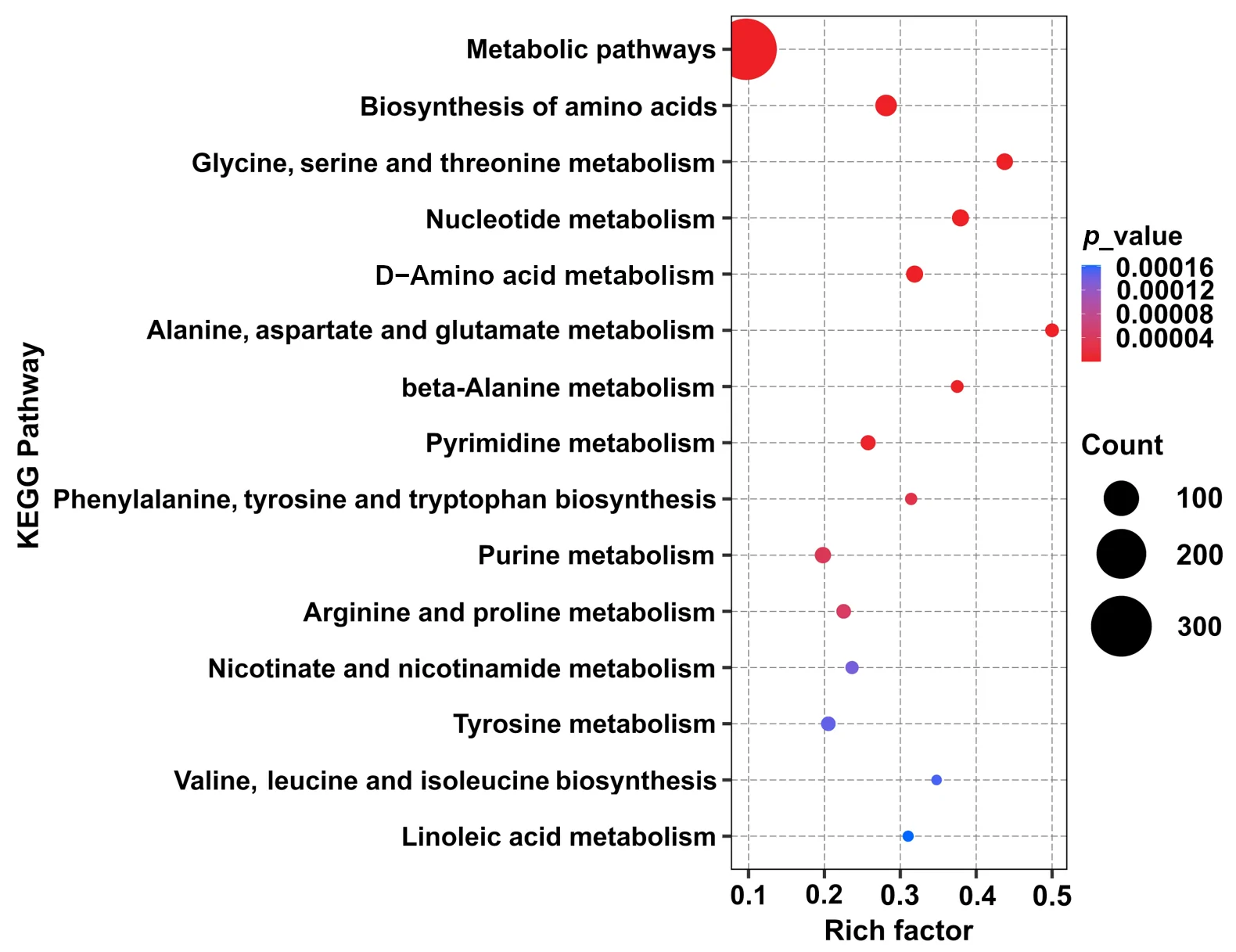
KEGG pathway enrichment analysis of differentially accumulated metabolites during tomato ripening. The size of the bubble represents the number of mapped metabolites, while its color indicates enrichment significance. Finally, the rich factor represents the proportion of metabolites associated with each pathway.

## Data Availability

The data presented in this study are available on request from the corresponding author.

## Supplementary Materials

The following supporting information can be downloaded at https://www.mdpi.com/article/10.3390/foods15193559/s1, Table S1: Tentatively identified compounds and their basic information; Table S2: Relative abundance (%) of tentatively identified compounds across different groups; Table S3: Correspondence between M1–M20 and the tentatively identified compounds in the VIP plot; Table S4: Correspondence between C1–C20 and the tentatively identified compounds in the correlation plot.
