# Supplementary material for: Ripening-Associated Changes in Physicochemical Properties and Metabolomic Profiles of Processing Tomato
Source: Foods. 2026 Oct 8;15(19):3559. doi: 10.3390/foods15193559 (PMC13665430; doi:10.3390/foods15193559)
Supplement: Supplementary file 1 [file foods-15-03559-s001.zip › foods-4560177-supplementary.pdf]

Supplementary Materials

Supplementary Tables S1-S4

Table S1. Tentatively identified compounds and basic information

The table lists the 78 GC-MS compounds included in the analysis, together with their CAS numbers, molecular weights, molecular formulas, and chemical classes.

No.	CAS No.	Compound name	MW	Retention Time（min）	Formula	Chemical class
1	106-42-3	p-Xylene	106.165	7.75	C8H10	Aromatic hydrocarbon
2	108-38-3	m-Xylene	106.165	7.94	C8H10	Aromatic hydrocarbon
3	1461-27-4	Isocarvestrene	136.234	8.69	C10H16	Terpene
4	3387-41-5	Sabinene	136.234	8.93	C10H16	Terpene
5	100-42-5	Styrene	104.149	10.1	C8H8	Aromatic hydrocarbon
6	192823-15-7	2,3,5,8-tetramethyldecane	198.388	13.41	C14H30	Aliphatic hydrocarbon
7	3391-86-4	Oct-1-en-3-ol	128.212	13.66	C8H16O	Alcohol
8	104-76-7	2-Ethylhexanol	130.228	14.35	C8H18O	Alcohol
9	3856-25-5	α-Copaene	204.351	14.34	C15H24	Terpene
10	432-25-7	β-Cyclocitral	152.233	16.75	C10H16O	Aldehyde
11	68832-35-9	trans-β-caryophyllene	204.351	17.07	C15H24	Terpene
12	119-36-8	Methyl salicylate	152.147	19.3	C8H8O3	Ester
13	112-39-0	Methyl palmitate	270.451	25.21	C17H34O2	Ester
14	29050-33-7	4(5)-carene	136.234	7.37	C10H16	Terpene
15	95-47-6	o-xylene	106.165	8.1	C8H10	Aromatic hydrocarbon
16	5989-27-5	Limonene	136.234	8.73	C10H16	Terpene
17	90-05-1	Guaiacol	124.140	20.5	C7H8O2	Phenol
18	66-25-1	Hexanal	100.159	6.77	C6H12O	Aldehyde
19	2548-87-0	(2E)-2-Octenal	126.196	13.31	C8H14O	Aldehyde
20	26001-58-1	(Z)-2-octen-1-ol	128.212	16.53	C8H16O	Alcohol
21	275-51-4	Azulene	128.171	18.75	C10H8	Aromatic hydrocarbon
22	25152-83-4	(E,Z)-2,4-decadien-1-al	152.233	19.11	C10H16O	Aldehyde
23	761-65-9	N,N-Dibutylformamide	157.253	19.13	C9H19NO	Nitrogen-containing compound
24	124-07-2	Octanoic acid	144.211	23.19	C8H16O2	Carboxylic acid
25	30086-02-3	(E,E)-3,5-octadien-2-one	124.180	15.04	C8H12O	Ketone

No.	CAS No.	Compound name	MW	Retention Time（min）	Formula	Chemical class
26	4312-99-6	1-Octen-3-one	126.196	10.97	C8H14O	Ketone
27	36431-60-4	1-Cyclopentene-1-carboxaldehyde, 5-ethyl-	124.180	13.2	C8H12O	Aldehyde
28	111-87-5	1-Octanol	130.228	15.53	C8H18O	Alcohol
29	2497-25-8	(Z)-2-decen-1-al	154.249	17.14	C10H18O	Aldehyde
30	5910-87-2	(2E,4E)-2,4-Nonadienal	138.207	18.06	C9H14O	Aldehyde
31	555-10-2	β-phellandrene	136.234	8.75	C10H16	Terpene
32	3777-69-3	2-Amylfuran	138.207	9.37	C9H14O	Heterocyclic compound
33	110-93-0	6-Methylhept-5-en-2-one	126.196	11.67	C8H14O	Ketone
34	111-11-5	Methyl octanoate	158.238	12.54	C9H18O2	Ester
35	2492-22-0	2,6-Dimethyl-2-trans-6-octadiene	138.250	12.85	C10H18	Terpene
36	18640-74-9	2-Isobutylthiazole	141.234	12.93	C7H11NS	Heterocyclic compound
37	1569-60-4	5-Hepten-2-ol,6-methyl	128.212	13.9	C8H16O	Alcohol
38	3796-70-1	Geranylacetone	194.313	20.31	C13H22O	Ketone
39	6125-24-2	Phenylnitroethane	151.163	24.18	C8H9NO2	Nitrogen-containing compound
40	1117-52-8	Farnesyl acetone	262.430	27.18	C18H30O	Terpene
41	106-26-3	(Z)-citral	152.233	17.71	C10H16O	Aldehyde
42	141-27-5	(E)-citral	152.233	18.99	C10H16O	Aldehyde
43	14901-07-6	β-Ionone	192.297	21.59	C13H20O	Ketone
44	111-27-3	1-Hexanol	102.175	11.94	C6H14O	Alcohol
45	539-52-6	Perillen	150.218	13.11	C10H14O	Heterocyclic compound
46	68039-49-6	2,4-Dimethyl-3-Cyclohexenecarboxaldehyde	138.207	15.19	C9H14O	Aldehyde
47	18829-56-6	(2E)-2-Nonenal	140.223	15.26	C9H16O	Aldehyde
48	18409-17-1	Trans-2-Octen-1-ol	128.212	16.47	C8H16O	Alcohol
49	25152-84-5	(2E,4E)-Deca-2,4-dienal	152.233	19.73	C10H16O	Aldehyde
50	53837-34-6	6,10-dimethylundeca-5,9-dien-2-ol	196.329	21.69	C13H24O	Alcohol
51	141-10-6	Pseudoionone	192.297	24.27	C13H20O	Ketone
52	97-53-0	Eugenol	164.201	24.72	C10H12O2	Phenol
53	1871-67-6	Trans-2-octenoic acid	142.196	24.88	C8H14O2	Carboxylic acid
54	7560-64-7	4-Methylcyclohex-3-enecarbaldehyde	124.180	13.19	C8H12O	Aldehyde
55	98678-70-7	2-Methyl-2-(4-methyl-3-pentenyl)-cyclopropanemethanol	168.276	18.08	C11H20O	Alcohol

No.	CAS No.	Compound name	MW	Retention Time（min）	Formula	Chemical class
56	79-77-6	4-(2,6,6-Trimethyl-1-cyclohexenyl)-3-buten-2-one	192.297	21.59	C13H20O	Ketone
57	55976-14-2	(Z)-1,4-Undecadiene	152.280	8.03	C11H20	Aliphatic hydrocarbon
58	71-41-0	1-Pentanol	88.148	10.09	C5H12O	Alcohol
59	1941-12-4	Phenol, 2-methoxy-3-(2-propenyl)	164.201	24.72	C10H12O2	Phenol
60	2511-91-3	1-Cyclopropylpentane	112.213	15.52	C8H16	Aliphatic hydrocarbon
61	143-08-8	1-Nonanol	144.254	17.22	C9H20O	Alcohol
62	14507-02-9	2,4-Decadien-1-ol	154.249	22.17	C10H18O	Alcohol
63	513-86-0	Acetoin	88.105	10.97	C4H8O2	Ketone
64	112-23-2	Heptyl formate	144.211	13.76	C8H16O2	Ester
65	2628-17-3	4-Vinylphenol	120.148	27.51	C8H8O	Phenol
66	60-12-8	Phenethyl alcohol	122.164	21.22	C8H10O	Alcohol
67	124-19-6	Nonanal	142.240	12.64	C9H18O	Aldehyde
68	60784-31-8	(Z)-2-nonen-1-al	140.223	15.24	C9H16O	Aldehyde
69	36258-08-9	Cyclopentanemethanol, 2-ethyl-rel-	128.210	16.03	C8H16O	Alcohol
70	230646-72-7	1,5,9-Decatriene, 2,3,5,8-tetramethyl-	192.340	17.29	C14H24	Terpene
71	90-02-8	Salicylaldehyde	122.121	17.8	C7H6O2	Aldehyde
72	5392-40-5	Citral	152.233	18.51	C10H16O	Aldehyde
73	2463-77-6	Undecenal	168.276	18.78	C11H20O	Aldehyde
74	106-25-2	cis-3,7-Dimethyl-2,6-octadien-1-ol	154.249	19.46	C10H18O	Alcohol
75	88116-46-5	1-Butylcyclohex-2-en-1-ol	154.249	21.72	C10H18O	Alcohol
76	3548-78-5	Pseudoionone	192.297	24.27	C13H20O	Ketone
77	1117-61-9	Citronellol	156.265	18.9	C10H20O	Alcohol

Table S2. Relative abundance of compounds across groups

Values are expressed as mean ± SD (n = 4 independent biological replicates per group). Non-detected compounds were assigned a value of zero before normalization of each sample to 100% of its total peak area.

No.	Compound	MG (%)	BR (%)	T (%)	P (%)	LR (%)	R (%)
1	p-Xylene	1.3 ± 1.5	1.3 ± 2.6	0.8 ± 1.0	0	0.2 ± 0.4	0
2	m-Xylene	2.3 ± 2.9	1.6 ± 2.1	1.6 ± 1.2	0.6 ± 0.3	0.4 ± 0.8	0.1 ± 0.2
3	Isocarvestrene	2.4 ± 2.8	0	0.2 ± 0.5	0	0	0
4	Sabinene	20 ± 4	0	0.5 ± 1.0	0	0	0
5	Styrene	35 ± 7	28 ± 5	18 ± 1	7.4 ± 1.0	9.5 ± 0.2	2.0 ± 0.2
6	2,3,5,8-tetramethyldecane	1.9 ± 1.3	0	0	0	0	0
7	Oct-1-en-3-ol	3.1 ± 0.7	7.4 ± 0.9	1.9 ± 0.1	0.3 ± 0.2	0.7 ± 0.5	0.4 ± 0.1
8	2-Ethylhexanol	0.2 ± 0.2	0	0.1 ± 0.1	0	0	0
9	α-Copaene	6 ± 4	10 ± 7	2.6 ± 0.6	0	0	0
10	β-Cyclocitral	1.5 ± 1.7	0	0	0.40 ± 0.04	0.2 ± 0.4	0.5 ± 0.2
11	trans-β-caryophyllene	0.8 ± 0.5	0	0	0	0	0
12	Methyl salicylate	7.3 ± 2.0	16 ± 3	0	0	0	0
13	Methyl palmitate	1.0 ± 1.2	0	1.9 ± 0.1	0.6 ± 0.1	0	0.9 ± 0.1
14	4(5)-carene	1.3 ± 1.6	0	0	0	0	0
15	o-xylene	4 ± 3	1.1 ± 1.3	0.5 ± 1.0	0.1 ± 0.2	0	0
16	Limonene	1.6 ± 1.9	0	0	0	0	0
17	Guaiacol	3 ± 3	0	1.0 ± 0.7	0.46 ± 0.03	1.22 ± 0.04	1.3 ± 0.1
18	Hexanal	0	6.5 ± 1.3	2.2 ± 0.1	2.2 ± 0.7	0.4 ± 0.5	0.6 ± 0.1
19	(2E)-2-Octenal	0	6.2 ± 1.5	2.9 ± 1.8	6.8 ± 0.8	3.2 ± 0.5	4.2 ± 0.5
20	(Z)-2-octen-1-ol	0	3.5 ± 0.5	0	0	0	0.2 ± 0.2
21	Azulene	0	4.5 ± 0.7	1.5 ± 1.0	0.45 ± 0.04	1.0 ± 0.7	0
22	(E,Z)-2,4-decadien-1-al	0	2.1 ± 1.5	0.5 ± 0.9	8.3 ± 0.5	8 ± 4	7.4 ± 2.2
23	N,N-Dibutylformamide	0.1 ± 0.2	0.2 ± 0.2	0	0	0	0
24	Octanoic acid	0	3.0 ± 0.6	2.5 ± 0.3	0.6 ± 0.2	0.5 ± 0.6	1.4 ± 0.6
25	(E,E)-3,5-octadien-2-one	0	2.0 ± 1.4	0	0	0	0
26	1-Octen-3-one	0	1.5 ± 1.7	0	0	0	0
27	1-Cyclopentene-1-carboxaldehyde, 5-ethyl-	0	1.0 ± 1.0	1.5 ± 1.0	0.2 ± 0.4	0	0.1 ± 0.2

No.	Compound	MG (%)	BR (%)	T (%)	P (%)	LR (%)	R (%)
28	1-Octanol	0	1.0 ± 1.2	0	0.2 ± 0.2	1.2 ± 2.5	3.6 ± 0.8
29	(Z)-2-decen-1-al	0	1.5 ± 1.7	0	0.3 ± 0.2	0	0.3 ± 0.2
30	(2E,4E)-2,4-Nonadienal	0	0.9 ± 1.0	0.8 ± 0.9	0	0	0.6 ± 0.1
31	β-phellandrene	0	0	1.6 ± 1.1	0	0	0
32	2-Amylfuran	0	0.6 ± 1.2	1.8 ± 0.2	1.2 ± 0.1	4.3 ± 0.1	1.6 ± 0.2
33	6-Methylhept-5-en-2-one	0	0	13 ± 1	20 ± 1	13 ± 1	11 ± 1
34	Methyl octanoate	0	0	3.9 ± 0.3	0.6 ± 0.1	1.4 ± 0.4	1.1 ± 0.2
35	2,6-Dimethyl-2-trans-6-octadiene	0	0	0.4 ± 0.1	0.5 ± 0.4	0	0
36	2-Isobutylthiazole	0	0	5.8 ± 0.9	2.0 ± 0.1	1.4 ± 0.3	2.8 ± 0.4
37	5-Hepten-2-ol,6-methyl	0	0	1.8 ± 0.1	3.1 ± 0.1	1.2 ± 0.8	1.1 ± 0.4
38	Geranylacetone	0	0.1 ± 0.2	22 ± 1	23 ± 2	19 ± 2	24 ± 1
39	Phenylnitroethane	0	0	0.8 ± 0.6	0	0	0.33 ± 0.03
40	Farnesyl acetone	0	0	1.2 ± 0.1	1.1 ± 0.1	0.9 ± 0.1	1.1 ± 0.2
41	(Z)-citral	0	0	0.7 ± 0.8	2.41 ± 0.04	0.5 ± 0.9	0.8 ± 0.6
42	(E)-citral	0	0	1.2 ± 0.8	5.3 ± 0.5	1.4 ± 0.5	0.4 ± 0.8
43	β-Ionone	0	0	0.5 ± 0.6	0.2 ± 0.3	0.7 ± 0.5	0.4 ± 0.5
44	1-Hexanol	0	0	0	0.9 ± 0.7	5.9 ± 0.6	4.7 ± 0.6
45	Perillen	0	0	0	0.83 ± 0.01	0	0.34 ± 0.03
46	2,4-Dimethyl-3-Cyclohexenecarboxaldehyde	0	0	0	0.37 ± 0.02	0	0.1 ± 0.1
47	(2E)-2-Nonenal	0	0	0	0.6 ± 0.4	0.2 ± 0.4	0.2 ± 0.3
48	Trans-2-Octen-1-ol	0	0	0	1.1 ± 0.5	5.7 ± 0.4	5.4 ± 0.9
49	(2E,4E)-Deca-2,4-dienal	0	0	0	3.5 ± 0.2	0	3.5 ± 2.6
50	6,10-dimethylundeca-5,9-dien-2-ol	0	0	0	0.45 ± 0.03	0	0
51	Pseudoionone	0	0	0	0.4 ± 0.3	0	0.1 ± 0.2
52	Eugenol	0	0	0	0.3 ± 0.4	0.4 ± 0.5	0.1 ± 0.2
53	Trans-2-octenoic acid	0	0	0	0.2 ± 0.2	1.0 ± 0.7	0.7 ± 0.5
54	4-Methylcyclohex-3-enecarbaldehyde	0	0	0.5 ± 1.1	0.4 ± 0.5	0	0
55	2-Methyl-2-(4-methyl-3-pentenyl)-cyclopropanemethanol	0	0	0	0.7 ± 0.6	0	0

No.	Compound	MG (%)	BR (%)	T (%)	P (%)	LR (%)	R (%)
56	4-(2,6,6-Trimethyl-1-cyclohexenyl)-3-buten-2-one	0	0	0.4 ± 0.8	0.2 ± 0.2	0.2 ± 0.5	0.4 ± 0.4
57	(Z)-1,4-Undecadiene	0	0	0	0.02 ± 0.03	0	0
58	1-Pentanol	0	0	0.2 ± 0.5	0.3 ± 0.3	1.7 ± 1.1	1.1 ± 0.1
59	Phenol, 2-methoxy-3-(2-propenyl)	0	0	0	0.3 ± 0.4	0	0.1 ± 0.2
60	1-Cyclopropylpentane	0	0	0	0.1 ± 0.2	3.6 ± 2.6	0
61	1-Nonanol	0	0	0	0	0.2 ± 0.2	0
62	2,4-Decadien-1-ol	0	0	0	0.1 ± 0.2	2.9 ± 1.0	3.9 ± 1.0
63	Acetoin	0	0	0	0	2.0 ± 1.5	1.6 ± 0.5
64	Heptyl formate	0	0	0	0	0.4 ± 0.5	0.5 ± 0.3
65	4-Vinylphenol	0	0	0	0	1.0 ± 1.4	0.2 ± 0.2
66	Phenethyl alcohol	0	0	0	0	0.6 ± 0.7	1.2 ± 0.8
67	Nonanal	0	0	0	0	0.05 ± 0.10	0.1 ± 0.1
68	(Z)-2-nonen-1-al	0	0.4 ± 0.8	0	0.2 ± 0.4	0	0.3 ± 0.4
69	Cyclopentanemethanol, 2-ethyl-rel-	0	0	0	0	0	0.44 ± 0.01
70	1,5,9-Decatriene, 2,3,5,8-tetramethyl-	0	0	0	0.04 ± 0.09	0	0.1 ± 0.1
71	Salicylaldehyde	0	0	0	0	0	0.42 ± 0.05
72	Citral	0	0	0.4 ± 0.7	0	0	1.6 ± 1.1
73	Undecenal	0	0	0	0	0	0.2 ± 0.2
74	cis-3,7-Dimethyl-2,6-octadien-1-ol	0	0	0	0	0	0.38 ± 0.04
75	1-Butylcyclohex-2-en-1-ol	0	0	0	0	0	0.3 ± 0.2
76	Pseudoionone	0	0	0	0.1 ± 0.3	0	0.3 ± 0.2
77	Citronellol	0	0	0	0	0	0.3 ± 0.2

Table S3. Mapping of M1-M20 in the VIP plot

M1-M20 follow the top-to-bottom order in the corresponding VIP plot. This table provides the label-to-feature mapping used to interpret the plot.

Code	Feature annotation (as in VIP data)
M1	2_O_methyl_4a_O_2S_3R_4S_5S_6R_3_4_5_trihydroxy_6_hydroxymethyl_oxan_2_yl_2S_4aR_6aR_6aS_6bR_8aR_9R_10R_11S_12aR_14bS_10_2S_3R_4R_5R_3_4_dihydroxy_5_2S_3R_4S_5S_6R_3_4_5_trihydroxy_6_hydroxymethyl_oxan_2_yl_oxyoxan_2_yl_oxy_11_hydroxy_9_hydroxymethyl_2_6a_6b_9_12a_pentamethyl_1_3_4_5_6_6a_7_8_8a_10_11_12_13_14b_tetradecahydropicene_2_4a_dicarboxylate
M2	6_6_carboxy_2_11_carboxy_4_hydroxymethyl_4_6a_6b_8a_11_14b_hexamethyl_14_oxo_2_3_4a_5_6_7_8_9_10_12_12a_14a_dodecahydro_1H_picen_3_yl_oxy_4_5_dihydroxy_tetrahydropyran_3_yl_oxy_3_4_5_trihydroxy_tetrahydropyran_2_carboxylic_acid
M3	Epicatechin_3_O_3_O_methylgallate
M4	Rhamnazin_3_isorhamninoside
M5	D_Glucopyranosiduronic_acid_3beta_16alpha_28_3_O_D_glucopyranosyl_2_O_3_4_hydroxyphenyl_1_oxopropyl_D_xylopyranosyl_oxy_16_hydroxy_28_oxoolean_12_en_3_yl
M6	methyl_3_ethenyl_4_2_3_4_9_tetrahydro_1H_pyrido_3_4_b_indol_1_ylmethyl_2_3_4_5_trihydroxy_6_hydroxymethyl_oxan_2_yl_oxy_3_4_dihydro_2H_pyran_5_carboxylate
M7	Hesperetin_7_O_glucoside
M8	5_hydroxy_2_4_hydroxyphenyl_7_2S_3R_4S_5S_6R_3_4_5_trihydroxy_6_hydroxymethyl_tetrahydropyran_2_yl_oxy_chroman_4_one
M9	4_4_Hydroxy_4_hydroxy_3_methoxyphenyl_methyl_3_hydroxymethyl_oxolan_2_yl_2_methoxyphenol
M10	5R_5aS_8aR_9R_5_8_8a_9_Tetrahydro_9_hydroxy_5_3_4_5_trimethoxyphenyl_furo_3_4_6_7_naphtho_2_3_d_1_3_dioxol_6_5aH_one
M11	Scytophycin_E
M12	2S_3R_4S_5R_6S_6_2_3_5_dihydroxy_4_methoxyphenyl_5_7_dihydroxy_4_oxochromen_3_yl_oxy_4_5_dihydroxy_2_methyloxan_3_yl_acetate
M13	1R_2S_6S_9S_10R_11R_14S_15S_18S_20S_23R_24S_6_10_23_trimethyl_20_2R_3R_4S_5S_6R_3_4_5_trihydroxy_6_hydroxymethyl_tetrahydropyran_2_yl_oxy_4_azahexacyclo_12_11_0_02_11_04_9_015_24_018_23_pentacosan_17_one
M14	3_Hexenyl_isovalerate_3Z
M15	2_3_4_Dihydroxyphenyl_5_7_dihydroxy_6_3_4_5_trihydroxy_6_hydroxymethyl_oxan_2_yl_2_3_dihydrochromen_4_one
M16	FR900359
M17	Haploside_C
M18	28_Glucosyl_3b_hydroxy_12_oleanene_30_methoxy_28_oic_acid_3_arabinosyl_1_3_glucuronide
M19	1S_3S_4S_10S_4_12_diacetyloxy_1_9_dihydroxy_15_2_hydroxy_3_2_methylbut_2_enoylamino_3_phenylpropanoyl_oxy_10_14_17_17_tetramethyl_11_oxo_6_oxatetracyclo_11_3_1_03_10_04_7_heptadec_13_en_2_yl_benzoate
M20	Cynarin

Table S4. Mapping of C1-C20 in the correlation plot

C1-C20 correspond to the labels used in the correlation plot. This table provides the label-to-feature mapping used to interpret the plot.

Code	Compound / feature name
C1	M425T222
C2	Gabexate
C3	Arg-Phe
C4	M423T162_2
C5	6-(2-(tert-Butyl)-5-(6-methylpyridin-2-yl)-1H-imidazol-4-yl)quinoxaline
C6	[(3aS,4S,5R,6R,8Z,10R,11aR)-5-acetyloxy-6-hydroxy-6,10-dimethyl-3-methylidene-2,7-dioxo-3a,4,5,10,11,11a-hexahydrocyclodeca[b]furan-4-yl] 2-methylprop-2-enoate
C7	Bms-378806
C8	M473T224_2
C9	2-Methyl-1,2,3,4-tetrahydro-beta-carboline
C10	Flavan-3,3′,4′,5,5′,7-hexol
C11	Pegamine
C12	Ethylphenylhydantoin
C13	erythro-(7R,8S)-Dadahol B
C14	Pyroglutamic acid
C15	1-Aminopropenal acetic acid
C16	6-Hydroxy-7-methyl-1-[3,4,5-trihydroxy-6-(hydroxymethyl)oxan-2-yl]oxy-1,4a,5,6,7,7a-hexahydrocyclopenta[c]pyran-4-carboxylic acid
C17	[(1R,2E,8S,10R,11S)-10,11-dihydroxy-6-(methoxymethyl)-1,10-dimethyl-5-oxo-4,14-dioxatricyclo[9.2.1.0^{3,7}]tetradeca-2,6-dien-8-yl] 2-methylprop-2-enoate
C18	5,7-Dihydroxy-3-[4-[3,4,5-trihydroxy-6-(hydroxymethyl)tetrahydropyran-2-yl]oxyphenyl]chromen-4-one
C19	5,6-Dimethyl-2,3-pyrazinedicarbonitrile
C20	Parishin E
